# cGAS exacerbates *Schistosoma japonicum* infection in a STING-type I IFN-dependent and independent manner

**DOI:** 10.1371/journal.ppat.1010233

**Published:** 2022-02-02

**Authors:** Le Liang, Yujuan Shen, Yuan Hu, Haipeng Liu, Jianping Cao

**Affiliations:** 1 National Institute of Parasitic Diseases, Chinese Center for Disease Control and Prevention, (Chinese Center for Tropical Diseases Research); Key Laboratory of Parasite and Vector Biology, National Health Commission of People’s Republic of China; World Health Organization Collaborating Center for Tropical Diseases, Shanghai, China; 2 Shanghai University of Medicine & Health Sciences, Shanghai, China; 3 The School of Global Health, Chinese Center for Tropical Diseases Research, Shanghai Jiao Tong University School of Medicine, Shanghai, China; 4 Clinical and Translational Research Center, Shanghai Pulmonary Hospital, School of Medicine, Tongji University, Shanghai, China; 5 Central Laboratory, Shanghai Pulmonary Hospital, School of Medicine, Tongji University, Shanghai, China; University of California Riverside, UNITED STATES

## Abstract

Schistosomiasis, which is caused by infection with *Schistosoma* spp., is characterized by granuloma and fibrosis in response to egg deposition. Pattern recognition receptors are important to sense invading *Schistosoma*, triggering an innate immune response, and subsequently shaping adaptive immunity. Cyclic GMP-AMP synthase (cGAS) was identified as a major cytosolic DNA sensor, which catalyzes the formation of cyclic GMP-AMP (cGAMP), a critical second messenger for the activation of the adaptor protein stimulator of interferon genes (STING). The engagement of STING by cGAMP leads to the activation of TANK-binding kinase 1 (TBK1), interferon regulatory factor 3 (IRF3), and the subsequent type I interferon (IFN) response. cGAS is suggested to regulate infectious diseases, autoimmune diseases, and cancer. However, the function of cGAS in helminth infection is unclear. In this study, we found that *Cgas* deficiency enhanced the survival of mice infected with *S*. *japonicum* markedly, without affecting the egg load in the liver. Consistently, *Cgas* deletion alleviated liver pathological impairment, reduced egg granuloma formation, and decreased fibrosis severity. In contrast, *Sting* deletion reduced the formation of egg granulomas markedly, but not liver fibrosis. Notably, *Cgas* or *Sting* deficiency reduced the production of IFNβ drastically in mice infected with *S*. *japonicum*. Intriguingly, intravenous administration of recombinant IFNβ exacerbated liver damage and promoted egg granuloma formation, without affecting liver fibrosis. Clodronate liposome-mediated depletion of macrophages indicated that macrophages are the major type of cells contributing to the induction of the type I IFN response during schistosome infection. Moreover, cGAS is important for type I IFN production and phosphorylation of TBK1 and IRF3 in response to stimulation with *S*. *japonicum* egg- or adult worm-derived DNA in macrophages. Our results clarified the immunomodulatory effect of cGAS in the regulation of liver granuloma formation during *S*. *japonicum* infection, involving sensing schistosome-derived DNA and producing type I IFN. Additionally, we showed that cGAS regulates liver fibrosis in a STING-type I–IFN-independent manner.

## Author summary

The sensing of invading pathogens by pattern recognition receptors (PRRs) is important for the host to mount an immune response. Cytosolic DNA receptor cGAS has been documented as critical for the induction of innate immunity, manifesting as a type I IFN response. However, little is known about the role of cGAS or type I IFN in the process of helminth infection. In this study, we identified an important role of the cGAS-STING-type I IFN signaling axis in driving schistosome infection-induced liver inflammation. Moreover, we revealed a hitherto unknown role of cGAS in the regulation of liver fibrosis during *Schistosoma* infection, a process that is independent of STING. Our study revealed cGAS as a novel functional receptor for the recognition of invading *Schistosoma*, paving the way for the development of novel strategies to treat schistosomiasis.

## Introduction

Schistosomiasis, which is caused by infection with *Schistosoma* spp., is ranked as the second most important parasitic disease from a public health perspective [1]. By conservative estimates, it afflicts at least 230 million people worldwide and is a global threat to human health [2]. The schistosomiasis pandemic in China is caused by infection with *Schistosoma japonicum* [3]. A pathological hallmark of schistosomiasis is the formation of granulomas and fibrosis in response to the deposition of eggs [4]. The innate immune system is critical for the discriminative recognition of self and non-self components. This system activates a cascade of signaling events in response to invading pathogens or danger signals to initiate innate immunity, which in turn plays an important role in shaping adaptive immunity to clear the infection and repair injury. Pathogen-associated molecular patterns (PAMPs), expressed by invading pathogens, are specifically recognized by corresponding pattern recognition receptors (PRRs), which are expressed by innate immune cells, such as dendritic cells and macrophages [5]. A series of PRRs, including Toll-like receptors (TLRs), C-type lectin receptors (CLRs), scavenger receptors (SRs), RIG-I like receptors (RLRs), Nod-like receptors (NLRs), and DNA receptors have been identified. The role of different PRRs in *S*. *japonicum* infection has been reported in related studies [6–12].

The detection of intracellular DNA has emerged as one of the important modes of innate immune activation [13]. At least 10 types of cytoplasmic DNA receptors have been identified and can be classified as stimulator of interferon genes (STING)-dependent or independent, according to whether they rely on the adaptor protein STING for downstream signaling [13,14]. The STING-dependent DNA receptors include cyclic guanosine monophosphate adenosine monophosphate synthase (cGAS), interferon gamma inducible protein 16 (IFI16), DNA-dependent activator of IFN-regulatory factors (DAI), and DEAD-box helicase 41 (DDX41) [14]. The identification of the cytosolic DNA sensor cGAS was a breakthrough in the field of DNA recognition and has become one of the most recognized and studied DNA receptors [15–18]. cGAS binds to dsDNA and catalyzes the formation of a second messenger, cyclic guanosine-adenosine monophosphate (cGAMP), which binds and activates the downstream STING protein. STING then recruits Tank binding kinase 1 (TBK1) and activates the transcription factor interferon regulatory factor 3 (IRF3), ultimately leading to the production of type I interferon and related immune factors [19].

The cGAS-STING pathway is involved in the development of infectious diseases, autoimmune diseases, and cancer [20,21]. Recently, a series of studies revealed that genomic DNA from protozoan parasites triggers the cGAS-STING pathway, and identified the positive and negative regulators that modulate signaling during parasite infection [22–27]. Induction of cGAS-STING-TBK1-IRF3 signaling has been implicated to promote the replication of *Toxoplasma gondii* [23]. Notably, a recent work demonstrated that cGAS/STING signaling is critical to mount an anti-*T*. *gondii* immune response in a mouse infection model, which was enhanced by the dense granule protein GRA15 secreted by *T*. *gondii* in a STING- and TNF receptor associated factor (TRAF)-dependent manner [24]. cGAS has been implicated as an important cytosolic sensor of *Plasmodium falciparum* genomic DNA and is critical for the induction of type I IFN in response to malaria parasites [25]. Oxidized DNA released from *Trypanosoma cruzi* extracellular vesicles signal the poly(ADP-ribose) polymerase 1 (PARP1)-cGAS-nuclear factor kappa B (NF-κB) pathway for proinflammatory macrophage activation and worsens the chronic inflammatory pathology in Chagas disease [26]. The cGAS-STING axis has been implicated in sensing *Leishmania donovani* DNA and activating the innate cytosolic surveillance pathway to promote parasite survival [27].

Although the function of cGAS-STING in protozoan infection has been characterized, little is known about the activation of this pathway in helminth infection. A recent work demonstrated that the STING signaling pathway is important for *S*. *mansoni* DNA sensing and the deficiency of these adaptor molecules led to enhanced resistance to infection [28]. However, the role of cGAS in the process of *Schistosoma* infection is largely unknown. During *Schistosoma* infection, the parasite is highly likely to release DNA into the host. Therefore, it would be intriguing to determine whether cGAS is critical for the sensing of parasite-derived DNA and the subsequent induction of the innate immune response to regulate the process of *Schistosoma japonicum* infection. In this study, we aimed to determine the role of cGAS and STING in sensing *Schistosom*a-derived DNA and regulating of the process of *S*. *japonicum* infection using both cellular and mouse infection models. We hope that the results will further our understanding of the pathogenesis of schistosomiasis and provide novel strategies for its treatment.

## Results

### cGAS exacerbates *S*. *japonicum* infection in mice

**Fig 1:**
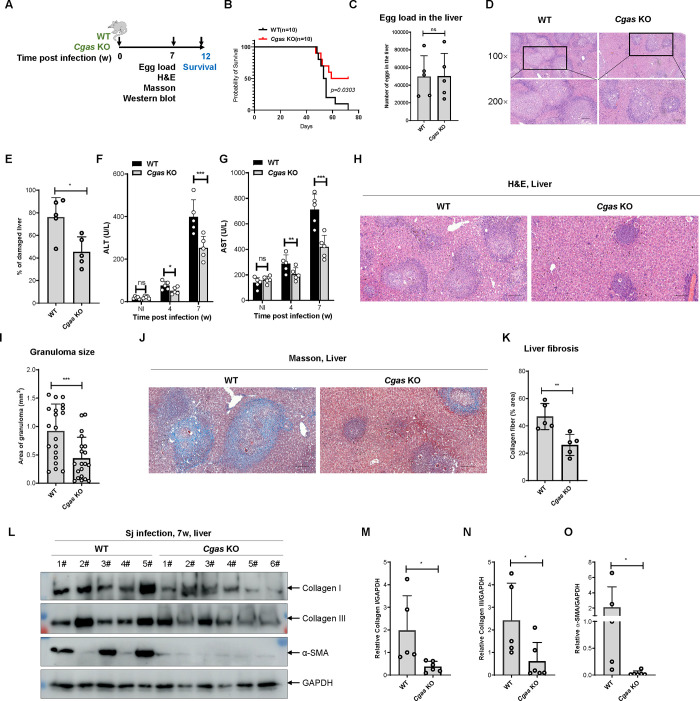
cGAS exacerbates *Schistosoma japonicum* infection in mice. (A) Scheme for monitoring of the survival of mice infected with *S*. *japonicum*. (B) The survival curve of mice infected with *S*. *japonicum*. The Kaplan–Meier method was used for the statistical analysis. (C) The egg loads in the livers of wild-type and *Cgas* knockout mice infected with *S*. *japonicum*. Data are expressed as the mean ± SD of the indicated number of mice from one of three independent experiments. (D-E) Representative imaging showing liver damage in the wild-type and *Cgas* knockout mice infected with *S*. *japonicum*. The quantification of the liver damage is shown in E. Data are expressed as the mean ± SD of the indicated number of mice from one of three independent experiments. (F-G) Measurement of the level of ALT (F) and AST in the sera of wild-type and *Cgas* knockout mice left uninfected or infected with *S*. *japonicum* for the indicated times. Data are expressed as mean ± SD of indicated number of mice from one of three independent experiments. (H-I) Representative imaging showing egg-induced granuloma in the livers of wild-type and *Cgas* knockout mice infected with *S*. *japonicum*. The quantification of the area of the granuloma is shown in (I). Data are expressed as the mean ± SD of the indicated number of granulomas. (J-K) Representative imaging showing Masson staining of the fibrosis in the livers of wild-type and *Cgas* knockout mice infected with *S*, *japonicum*. The quantification of the area of fibrosis is shown in (K). Data are expressed as the mean ± SD of the indicated number of mice from one of three independent experiments. (L-O) Western blotting of the indicated fibrosis-related proteins in the livers of wild-type and *Cgas* knockout mice infected with *S*. *japonicum*. The quantification of gray intensity is shown in (M-O). An unpaired Student’s *t*-test was used for the statistical analysis in (C, E, I, K and M-O). Two-way ANOVA with Bonferroni’s post hoc test was used for the statistical analysis in (F and G). ns, not significant; *, *p* < 0.05; **, *p* < 0.01; ***, *p* < 0.001. Scale bar, 200 μm.

To clarify the function of cGAS in the process of *S*. *japonicum* infection, we employed an acute infection model by infecting mice with schistosome cercariae [10,29]. Both wild-type and *Cgas*^-/-^ mice on a C57/BL6 background were infected with cercariae and the survival of the mice was monitored (Fig 1A). Both groups of mice began to die at around 50 days post-infection. The deletion of *Cgas* significantly enhanced the survival rate of the mice, indicating that cGAS promoted the process of *S*. *japonicum* infection in mice (Fig 1B).

*S*. *japonicum* infection of mice causes liver egg deposition and induces egg granuloma formation, causing pathological damage to the liver [30–32]. Next, we infected wild-type and *Cgas*^-/-^ mice with cercariae and isolated their livers at week 7 post-infection for further analysis. The egg loads in the livers of wild-type and *Cgas*^-/-^ mice were not significantly different (Fig 1C), indicating that deficiency of cGAS did not affect egg deposition in the liver after *S*. *japonicum* infection. Hematoxylin and eosin (H&E) staining of liver sections revealed that *S*. *japonicum* infection caused pathological damage to the liver of mice (Fig 1D). By quantifying the area of pathological impairment, the proportions of pathological damage in the liver of wild-type and *Cgas*^-/-^ mice were determined as 76.20 ± 17.20% and 45.40 ± 13.30%, respectively (p = 0.0132) (Fig 1E), suggesting that cGAS exacerbates the pathological liver damage caused by *S*. *japonicum* infection. Consistently, infection by *S*. *japonicum* in mice caused impaired liver function, as manifested by increased levels of aspartate aminotransferase (AST) and alanine aminotransferase (ALT) (Fig 1F and 1G), while the deletion of *Cgas* markedly reversed the impairment of liver function (Fig 1F and 1G). We then observed the size of granulomas formed by individual eggs and the quantitative data showed that the sizes of granulomas of eggs in the liver of wild-type and *Cgas*^-/-^ mice were 0.9205 ± 0.4383 mm^2^ and 0.4762 ± 0.3718 mm^2^, respectively (p = 0.0010) (Fig 1H and 1I), suggesting that cGAS promotes egg-induced granuloma formation during *S*. *japonicum* infection.

Another important feature of *S*. *japonicum* infection is liver fibrosis [33]. Masson staining of liver tissue sections revealed that marked fibrosis occurred around the deposited eggs in the liver (Fig 1J). Quantification of liver fibrosis showed that the proportion of liver fibrosis in wild-type versus *Cgas*^-/-^ mice was 46.80 ± 9.524% *vs*. 26.00 ± 7.714%, respectively (p = 0.0053) (Fig 1K), suggesting that cGAS promotes liver fibrosis formation during *S*. *japonicum* infection. We then extracted liver tissue proteins and detected the expression of fibrosis-related indicators, including Collagen I, Collagen III, and alpha smooth muscle actin (αSMA), using western blotting. The results demonstrated that the levels of liver fibrosis-related indicators were significantly downregulated after deletion of *Cgas* (Fig 1L–1O), suggesting that cGAS could aggravate liver fibrosis caused by *S*. *japonicum* infection.

### STING exacerbates *S*. *japonicum* infection in mice

**Fig 2:**
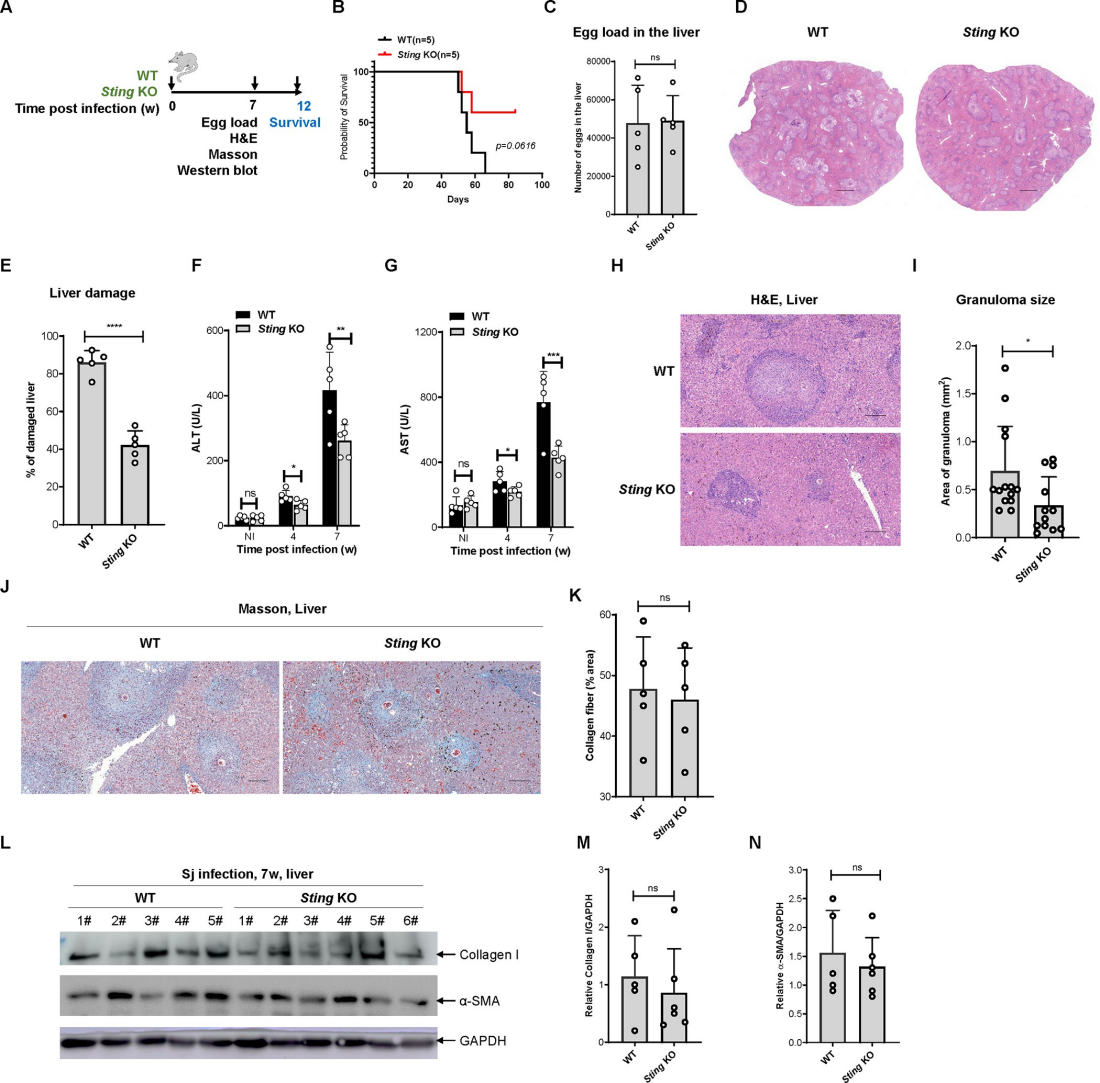
STING exacerbates *Schistosoma japonicum* infection in mice. (A) The scheme for monitoring of the survival of wild-type and *Sting* knockout mice infected with *S*. *japonicum*; (B) The survival curve of wild-type and *Sting* knockout mice infected with *S*. *japonicum*. (C) The egg loads in the livers of wild-type and *Sting* knockout mice infected with *S*. *japonicum*. Data are expressed as the mean ± SD of the indicated number of mice from one of three independent experiments. (D-E) Representative imaging showing liver damage in the wild-type and *Sting* knockout mice infected with *S*. *japonicum*. Scale bar, 1 000 μm. The quantification of the liver damage is shown in E. Data are expressed as the mean ± SD of the indicated number of mice from one of three independent experiments. (F-G) Measurement of the level of ALT (F) and AST in the sera of wild-type and *Sting* knockout mice left uninfected or infected with *S*. *japonicum* for the indicated times. Data are expressed as mean ± SD of indicated number of mice from one of three independent experiments. (H-I) Representative images showing egg-induced granulomas in the livers of wild-type and *Sting* knockout mice infected with *S*. *japonicum*. Scale bar, 200 μm. The quantification of the area of the granuloma is shown in (I). Data are expressed as the mean ± SD of the indicated number of granulomas from one of three independent experiments. (J-K) Representative images showing Masson staining of the fibrosis in the livers of wild-type and *Sting* knockout mice infected with *S*. *japonicum*. Scale bar, 200 μm. The quantification of the area of fibrosis is shown in (K). Data are expressed as the mean ± SD of the indicated number of mice from one of three independent experiments. (L-N) Western blotting detection of the indicated fibrosis-related proteins in the livers of wild-type and *Sting* knockout mice infected with *Schistosoma japonicum*. The quantification of gray intensity is shown in (M and N). An unpaired Student’s t-test was used for the statistical analysis in (C, E, I, K, M and N). Two-way ANOVA with Bonferroni’s post hoc test was used for the statistical analysis in (F and G). ns, not significant; *, *p* < 0.05; **, *p* < 0.01; ***, *p* < 0.001.

STING is the downstream adaptor protein responsible for the sensing of cGAMP catalyzed by cGAS upon recognition of cytosolic DNA [21]. Therefore, we next examined the functional role of STING in the process of *S*. *japonicum* infection (Fig 2A). By infecting both wild-type and *Sting*^-/-^ mice with cercaria and monitoring of their mortality, we observed that the deletion of *Sting* moderately, but not significantly, affected the survival of mice post infection (Fig 2B). Notably, the egg loads in the liver of wild-type and *Sting*^-/-^ mice were not significantly different, indicating that *Sting* knockout did not affect egg deposition in the liver after *S*. *japonicum* infection (Fig 2C). Histological analysis of liver sections revealed that the deletion of *Sting* caused a marked reduction in the pathological damage to the livers of infected mice (Fig 2D and 2E), suggesting that STING exacerbates the pathological damage to the liver caused by *S*. *japonicum* infection. Consistently, the deletion of *Sting* ameliorated schistosome infection-induced damage to liver function, as demonstrated by reduced levels of ALT and AST (Fig 2F and 2G). Moreover, the data showed that *Sting* deficiency significantly reduced the size of the egg granulomas in the liver (Fig 2H and 2I), suggesting that STING promotes egg-induced granuloma formation during *S*. *japonicum* infection. Furthermore, Masson staining of liver tissue sections demonstrated that the proportion of liver fibrosis in wild-type versus *Sting*^-/-^ mice was comparable (Fig 2J and 2K). Consistently, deficiency of *Sting* did not significantly affect the abundance of fibrosis-related indicators, including Collagen I and αSMA in the liver of mice infected with *S*. *japonicum* for 7 weeks (Fig 2L–2N). These results indicated that STING is not involved in the formation of liver fibrosis during *S*. *japonicum* infection, which is distinct from the function of cGAS.

### Schistosoma infection induces a type I IFN response

**Fig 3:**
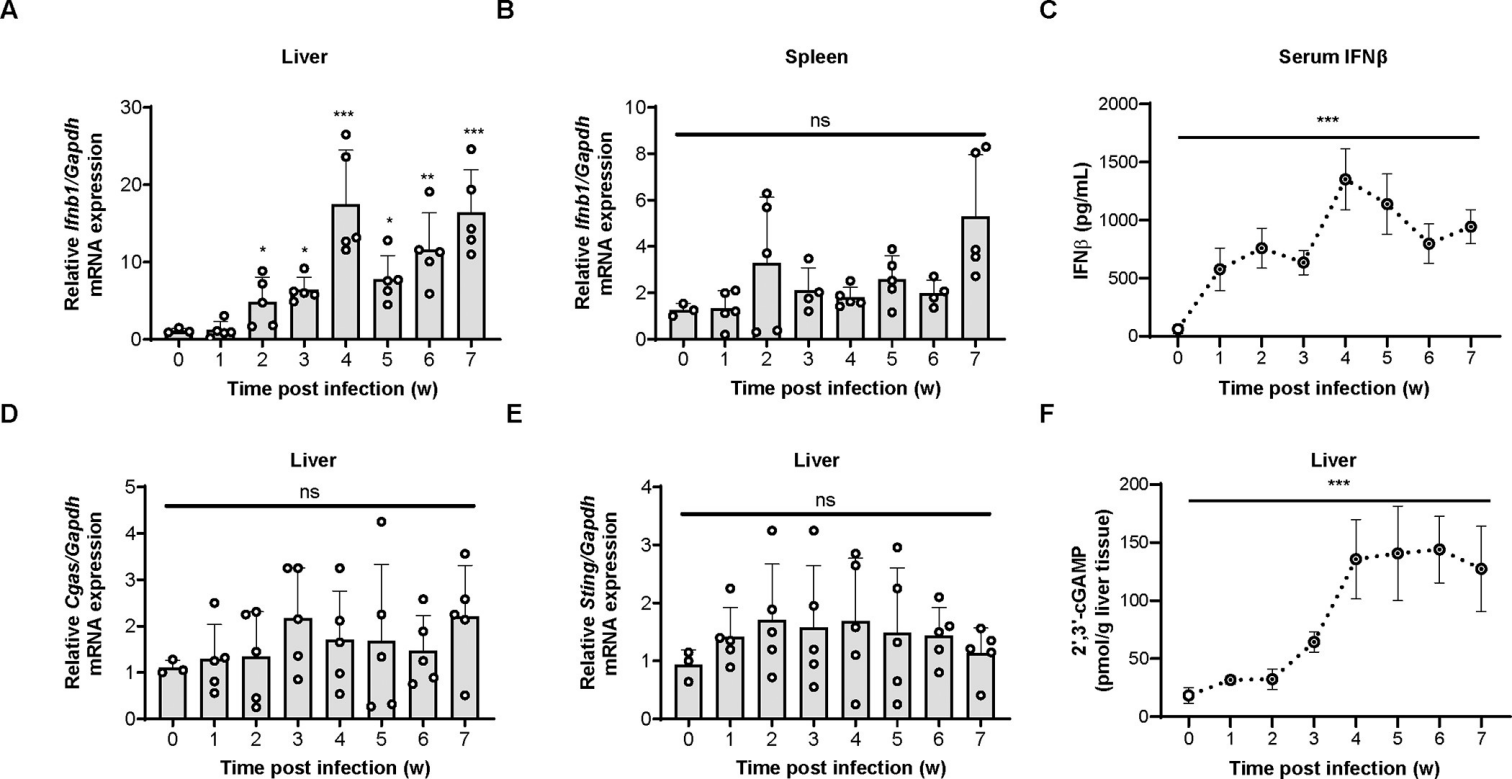
*Schistosoma* infection induces a type I IFN response. (A-B) qRT-PCR measurement of *Ifnb1* transcripts in the liver (A) and lung tissues (B) of mice infected with *S*. *japonicum* for the indicated times. (C) ELISA detection of IFNβ in serum harvested from mice infected with *S*. *japonicum* for the indicated times. (D-E) qRT-PCR measurement of the transcripts of *Cgas* (D) and *Sting* (E) in the liver tissues of mice infected with *S*. *japonicum* for the indicated times. (F) Measurement of the abundance of cGAMP in the liver tissues of mice infected with *S*. *japonicum* for the indicated times using a cGAMP Enzyme Immunoassay Kit. One-way ANOVA with Bonferroni’s post hoc test were used for the statistical analysis. ns, not significant; *, *p* < 0.05; **, *p <* 0.01, ***, *p <* 0.001.

The cGAS-STING signaling pathway activates the type I interferon immune response, and one of the important effector proteins is IFNβ [19,20]. However, the regulatory role of IFNβ in the process of *S*. *japonicum* infection remains elusive. We established an acute mouse model of *S*. *japonicum* infection and measured the expression level of *Ifnb1* in the liver and spleen using quantitative real-time reverse transcription PCR (qRT-PCR). The results showed that the expression level of *Ifnb1* mRNA in liver tissues increased significantly within 4 weeks of schistosome infection, and then remained at a relatively stable level (Fig 3A). However, the *Ifnb1* mRNA expression levels in spleen tissues were relatively low and no induction was observed in response to *S*. *japonicum* infection (Fig 3B). We then collected peripheral blood by sacrificing mice at each week post infection and measured the level of IFNβ in the serum using an enzyme-linked immunosorbent assay (ELISA). The results demonstrated that *S*. *japonicum* infection caused a significant increase in the abundance of the IFNβ protein as early as one week after infection, with IFNβ levels peaking at week 4 post infection and then gradually decreasing and remaining at a certain level (Fig 3C).

We then determined whether the induction of a type I IFN response to schistosome infection is caused by increased expression of cGAS and STING. qRT-PCR measurement of the transcripts of *Cgas* and *Sting* in the liver of schistosome-infected mice demonstrated that *S*. *japonicum* infection did not affect *Cgas* and *Sting* mRNA levels significantly (Fig 3D and 3E), although the induction of type I IFN has been previously demonstrated to exert positive feedback control of the expression of cGAS and STING [23,34]. Intriguingly, an ELISA measurement of the abundance of cGAMP in the homogenates of liver revealed that schistosome infection caused an elevation of cGAMP in the liver, which reached a peak at 4 weeks post infection (Fig 3F), coincident with the expression pattern of IFNβ in the liver (Fig 3A and 3C). These results indicated that schistosome infection triggers the type I IFN response, which involves the generation of cGAMP in the liver.

### IFNβ exacerbates *S*. *japonicum* infection in mice

**Fig 4:**
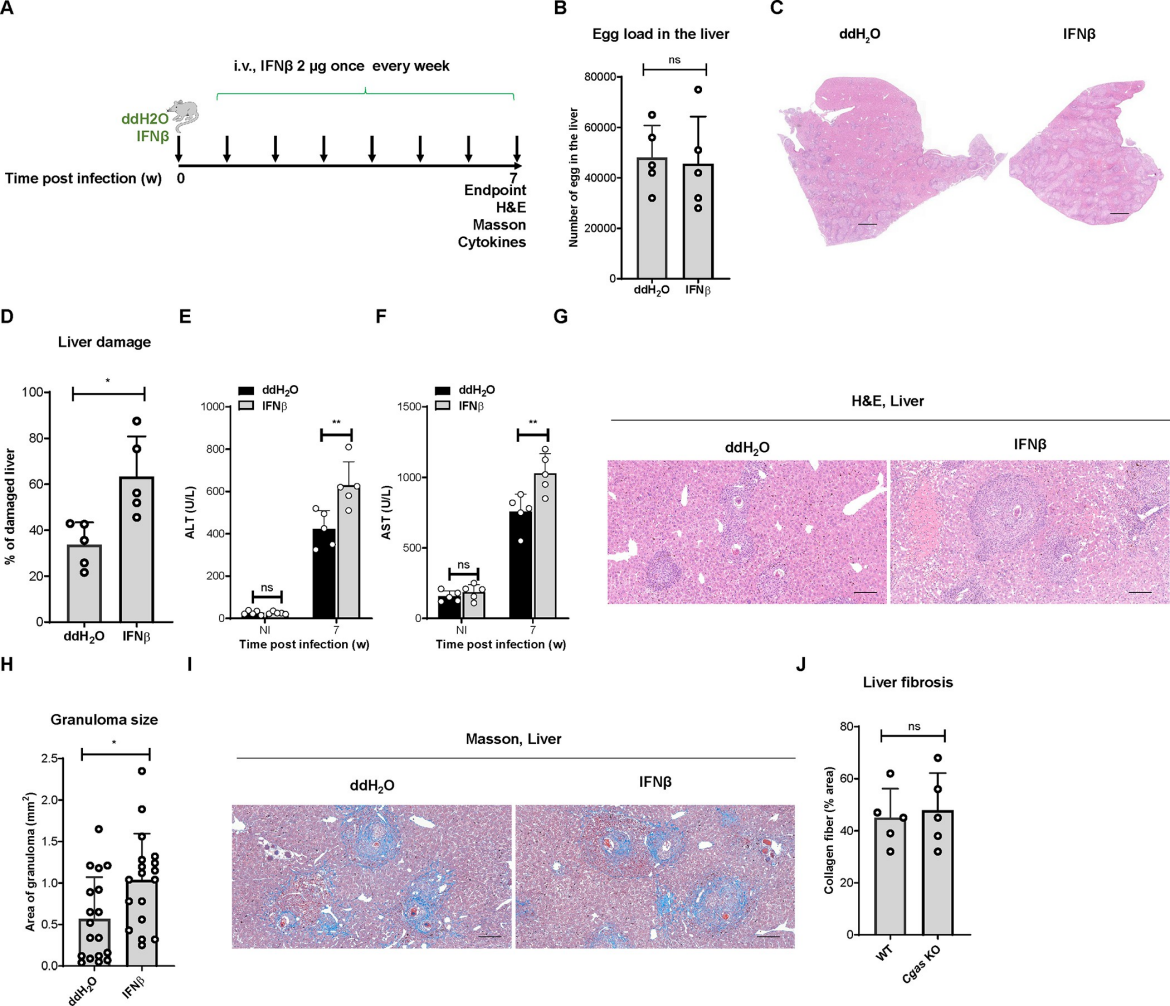
IFNβ exacerbates *Schistosoma japonicum* infection in mice. (A) Schematic diagram showing the experimental procedure for the tail vein injection of phosphate-buffered saline (PBS) control and 2 μg of recombinant interferon beta once a week for 7 weeks in mice infected with *S*. *japonicum*. The liver tissues were harvested for further experiments. (B) The egg loads in the liver of mice infected with *S*. *japonicum* left untreated or treated with interferon beta. Data are expressed as the mean ± SD of the indicated number of mice from one of three independent experiments. (C-D) Representative images showing liver damage in mice infected with *S*. *japonicum* left untreated or treated with interferon beta. Scale bar, 1 000 μm. The quantification of the liver damage is shown in D. Data are expressed as the mean ± SD of the indicated number of mice from one of three independent experiments. (E-F) Measurement of the level of ALT (E) and AST (F) in the sera of mice infected with *S*. *japonicum* left untreated or treated with interferon beta by tail vein injection. Data are expressed as the mean ± SD of the indicated number of mice from one of three independent experiments. (G-H) Representative imaging showing egg-induced granulomas in the livers of mice infected with *S*. *japonicum* left untreated or treated with interferon beta by tail vein injection. Scale bar, 200 μm. The quantification of the area of the granulomas is shown in (H). Data are expressed as the mean ± SD of indicated number of granulomas of mice from one of three independent experiments. (I-J) Representative images showing Masson staining of the fibrosis in the liver of mice infected with *S*. *japonicum* left untreated or treated with interferon beta. Scale bar, 200 μm. The quantification of the area of fibrosis is shown in (J). Data are expressed as the mean ± SD of the indicated number of mice from one of three independent experiments. An unpaired Student’s t-test was used for the statistical analysis in (B, D, H and J). Two-way ANOVA with Bonferroni’s post hoc test was used for the statistical analysis in (E and F). ns, not significant; *, *p* < 0.05; **, *p* < 0.01.

*S*. *japonicum* infection induces a type I interferon response; therefore, we further investigated whether type I interferon influences the process of *S*. *japonicum* infection. Administration of recombinant IFNβ by tail vein injection every week post infection did not significantly affect the egg loads in the liver (Fig 4A and 4B), indicating that IFNβ levels did not affect the deposition of eggs in the liver after *S*. *japonicum* infection. However, the administration of IFNβ markedly increased the pathological damage in the liver (Fig 4C and 4D) and impaired liver function, as indicated by the AST and ALT levels (Fig 4E and 4F), indicating that IFNβ promotes the induction of pathological liver damage of mice infected with *S*. *japonicum*. Moreover, quantification of the size of the granulomas formed by individual eggs in the liver tissue sections demonstrated that IFNβ promoted egg-induced granuloma formation during *S*. *japonicum* infection (Fig 4G and 4H). Considering that cGAS, but not STING, promotes *S*. *japonicum* infection-induced liver fibrosis, we then investigated whether IFNβ affects this process. Quantification of liver fibrosis demonstrated that the proportions of liver fibrosis in the control and IFNβ complemented mice were 45.00 ± 11.16% and 47.80 ± 14.39%, respectively (p = 0.7399) (Fig 4I and 4J), suggesting that IFNβ does not affect liver fibrosis formation during *S*. *japonicum* infection.

### cGAS-STING is essential for the *S*. *japonicum* infection-induced type I IFN response

**Fig 5:**
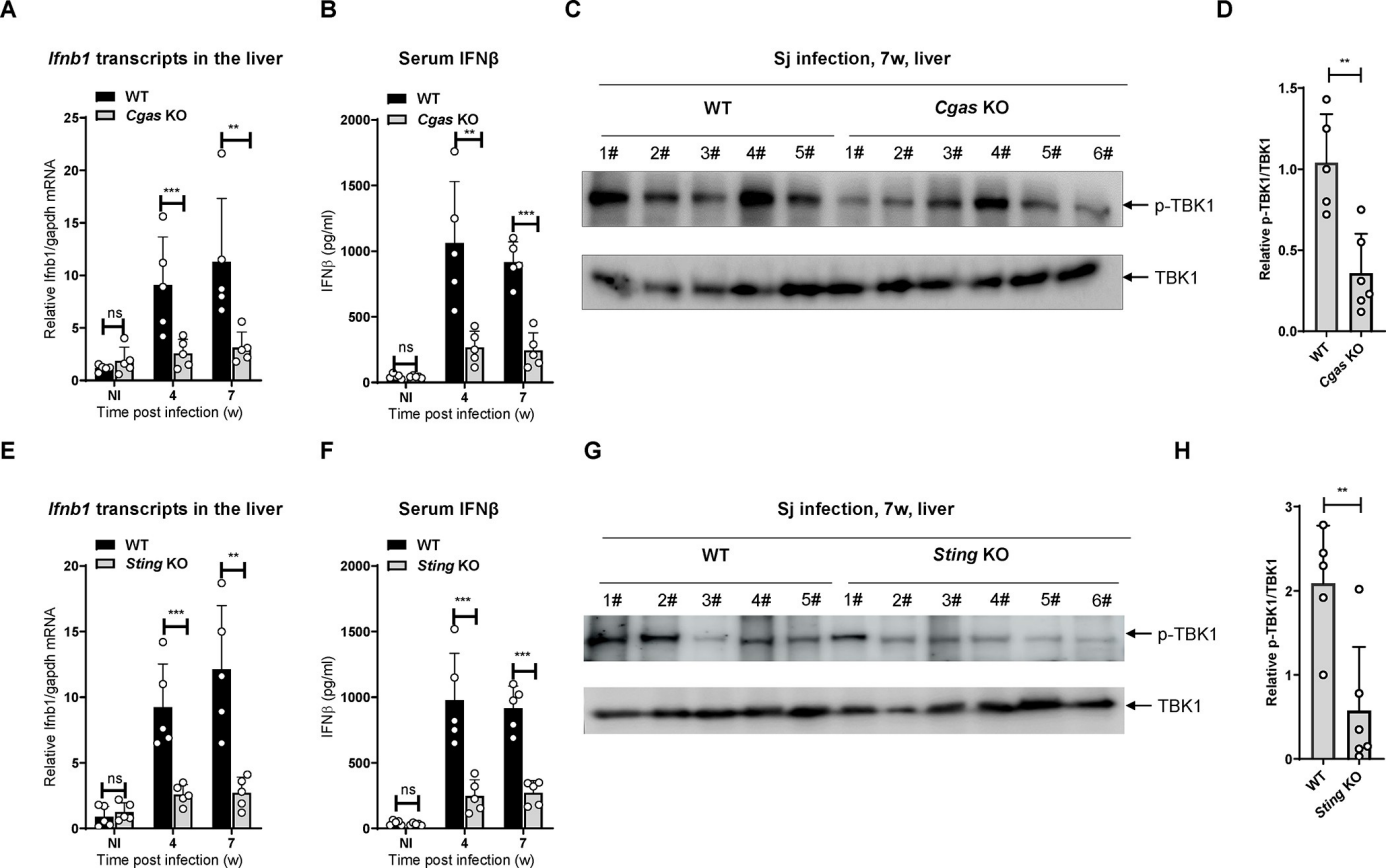
The cGAS-STING axis is essential for *Schistosoma japonicum* infection-induced type I IFN response. (A) qRT-PCR measurement of *Ifnb1* transcripts in the liver of wild-type and *Cgas* KO mice infected with *S*. *japonicum* for the indicated times (B) ELISA detection of IFNβ in the serum of wild-type and *Cgas* KO mice infected with *S*. *japonicum* for the indicated times. (C-D) Western blotting detection of the phosphorylation of TBK1 in the liver of wild-type and *Cgas* KO mice infected with *Schistosoma japonicum* for 7 weeks. (D) The quantification of the gray intensity of pTBK1 is shown in (D). (E) qRT-PCR measurement of *Ifnb1* transcripts in the liver of wild-type and *Sting* KO mice infected with *S*. *japonicum* for the indicated times. (F) ELISA detection of IFNβ in the serum of wild-type and *Sting* KO mice infected with *S*. *japonicum* for the indicated times. (G-H) Western blotting detection of the phosphorylation of TBK1 in the liver of wild-type and *Sting* KO mice infected with *S*. *japonicum* for 7 weeks. The quantification of the gray intensity of pTBK1 is shown in (H). Two-way ANOVA with Bonferroni’s post hoc test were used for the statistical analysis in (A, B, E and F). An unpaired Student’s t-test was used for the statistical analysis in (D and H). ns, not significant; *, *p* < 0.05, **, *p <* 0.01, ***, *p <* 0.001. Scale bar, 200 μm.

To investigate whether cGAS affects the type I interferon response during *S*. *japonicum* infection, we measured the mRNA levels of *Ifnb1* in liver tissues and IFNβ in peripheral blood from wild-type mice and *Cgas* knockout (KO) mice at 4 and 7 weeks post-infection, respectively. The results showed that the deficiency of *Cgas* markedly reduced the *Ifnb1* transcripts in the liver and IFNβ in the blood at both time points post infection (Fig 5A and 5B), indicating that cGAS is important for the induction of IFNβ during *S*. *japonicum* infection. To further investigate the effect of cGAS on the type I interferon response during *S*. *japonicum* infection, we detected the levels of the phosphorylated signaling molecule TBK1 in the liver tissues from wild-type mice and *Cgas* KO mice infected for 7 weeks. The results showed that the level of phosphorylated TBK1 decreased significantly when *Cgas* was knocked out (Fig 5C and 5D), indicating that cGAS is critical for TBK1 phosphorylation induced by *S*. *japonicum* infection.

We then determined whether STING affects the type I interferon response during *S*. *japonicum* infection in a way similar to cGAS. The results demonstrated that the levels of *Ifnb1* transcripts in the liver and IFNβ in the blood in the *Sting* KO mice were much less than those in wild-type mice at both 4 and 7 weeks post infection (Fig 5E and 5F), indicating that STING is required for the induction of IFNβ during *S*. *japonicum* infection. Moreover, the deletion of *Sting* marked reduced the level of phosphorylated TBK1 (Fig 5G and 5H), indicating that STING is also critical for TBK1 phosphorylation induced by *S*. *japonicum* infection.

### Sensing of schistosome-derived DNA by cGAS in macrophages mediates the type I IFN response to *S*. *japonicum* infection

**Fig 6:**
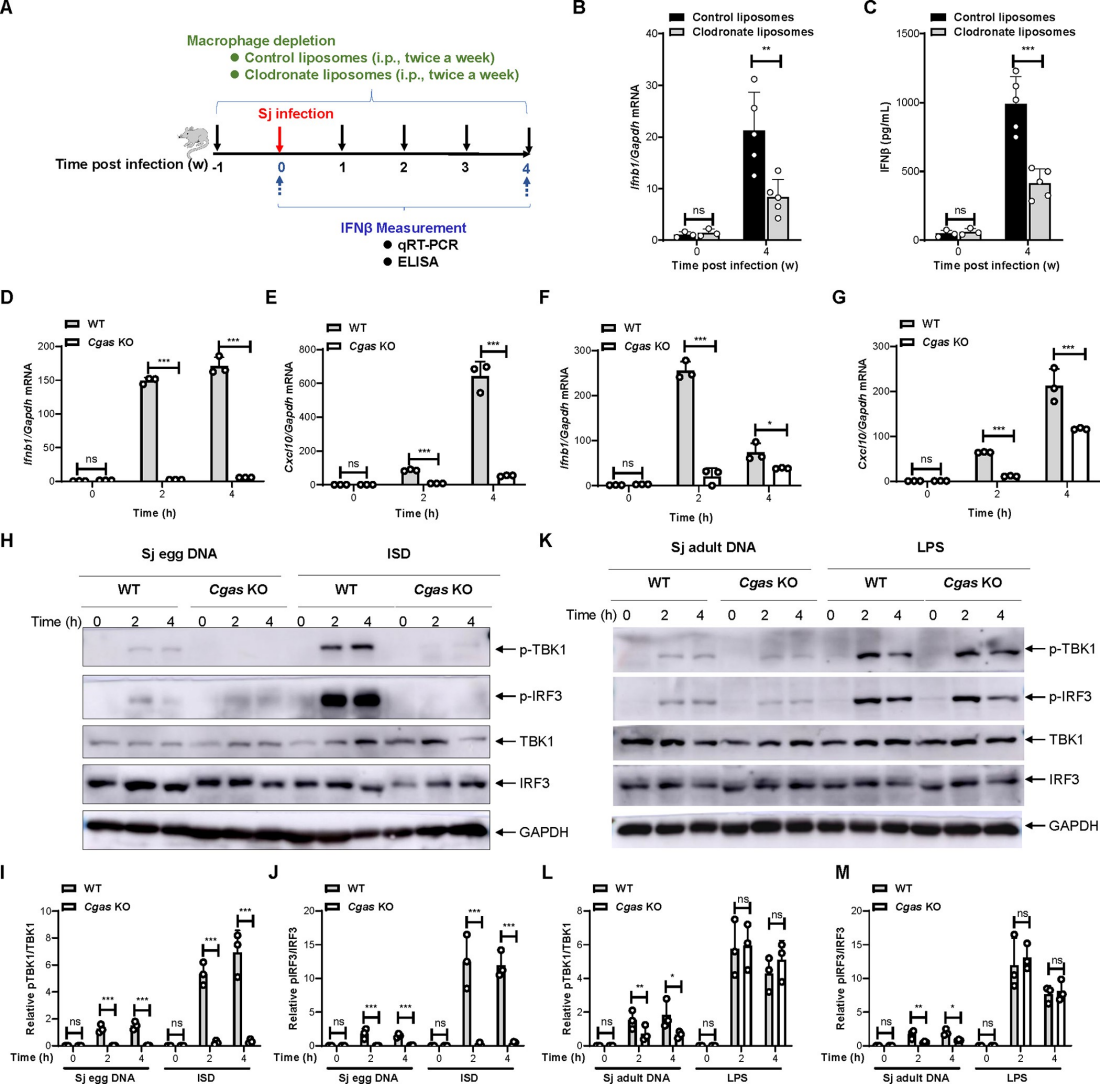
Sensing of schistosome-derived DNA by cGAS in macrophages mediates type I IFN response to *S*. *japonicum* infection. (A) Scheme for the clodronate liposome-mediated depletion of macrophages in mice and detection of interferon beta in the liver and sera of mice infected with *S*. *japonicum*. Mice were treated with control liposomes or clodronate liposomes twice a week for 1 week before infection and administered continuously for 4 weeks post infection at an interval of twice a week. The liver tissue and sera were collected from mice left uninfected or infected with *S*. *japonicum* for 4 weeks for further detection of interferon beta. (B) qRT-PCR measurement of *Ifnb1* transcripts in the liver tissues of mice infected with *S*. *japonicum* for the indicated times treated with control or clodronate liposomes. (C) ELISA detection of IFNβ in serum harvested from mice infected with *S*. *japonicum* for the indicated times treated with control or clodronate liposomes. (D-E) qRT-PCR measurement of transcripts of *Ifnb1* and *Cxcl10* in wild-type and *Cgas* knockout peritoneal macrophages transfected with DNA from egg of *S*. *japonicum* for the indicated times. (F-G) qRT-PCR measurement of the transcripts of *Ifnb1* and *Cxcl10* in wild-type and *Cgas* knockout peritoneal macrophages transfected with DNA from adult schistosomes for the indicated times. (H-J) Western blotting detection of the indicated proteins in the lysates of mouse peritoneal macrophages isolated from wild-type and *Cgas* KO mice stimulated with *S*. *japonicum* egg DNA and ISD for the indicated times (H). The quantification data is shown in (I and J). (K-M) Western blotting detection of the indicated proteins in the lysates of mouse peritoneal macrophages isolated from wild-type and *Cgas* KO mice stimulated with adult *S*. *japonicum* DNA and LPS for the indicated times (K). The quantification data is shown in (L and M). Two-way ANOVA with Bonferroni’s post hoc test were used for the statistical analysis in (D-G). ns, not significant; *, *p* < 0.05, **, *p <* 0.01, ***, *p <* 0.001.

Studies have shown that macrophages are involved in the process of *S*. *japonicum* infection, and in particular, macrophages can reshape the adaptive immune response by regulating cytokine secretion [35–37]. To clarify whether macrophages are the cellular origin of type I IFN during schistosome infection, we depleted macrophages using clodronate liposomes [38]. Control liposomes and clodronate liposomes were injected twice a week before cercariae infection and continually injected twice a week for another 4 weeks post infection (Fig 6A). The administration of clodronate liposomes markedly reduced the level of *Ifnb1* transcripts in the liver and the abundance of IFNβ in the sera of mice infected with *S*. *japonicum* for 4 weeks (Fig 6B and 6C). These data indicated that macrophages are the major type of cells contributing to the induction of the type I IFN response during schistosome infection.

We next assessed whether cGAS is engaged by schistosome-derived DNA, which thereby triggers the type I IFN response. We employed immunostimulatory DNA (ISD), a ligand of cGAS, and lipopolysaccharide (LPS), a ligand of TLR4, as positive and negative controls for the stimulation of wild-type and *Cgas* KO mouse peritoneal macrophages. As expected, the data demonstrated that deficiency of cGAS completely abrogated the transcription of *Ifnb1* and *Cxcl10* (encoding C-X-C motif chemokine ligand 10) in response to stimulation with ISD (S1A and S1B Fig) but not to LPS (S1C and S1D Fig). We then purified *S*. *japonicum* eggs and extracted egg DNA, transfected wild-type and *Cgas* KO peritoneal macrophages with the DNA, and measured the transcripts of *Ifnb1* and *Cxcl10* using qRT-PCR. The results showed that egg DNA induced a type I interferon response, while the transcription of *Ifnb1* and *Cxcl10* was largely abrogated when *Cgas* was knocked out (Fig 6D and 6E). Moreover, we isolated the adult schistosomes and purified DNA for the stimulation of macrophages. Similar to schistosome egg-derived DNA, the adult worm-derived DNA induced robust expression of *Ifnb1* and *Cxcl10* (Fig 6F and 6G). Importantly, deficiency of *Cgas* markedly reduced the transcription of *Ifnb1* and *Cxcl10*; however, this process was not completely abrogated (Fig 6F and 6G). These results indicated that cGAS plays an important role in the induction of the type I interferon response through recognition of schistosome-derived DNA during the process of *S*. *japonicum* infection.

We then investigated whether cGAS affects egg DNA-induced activation of signaling pathways. We transfected wild-type and *Cgas* KO peritoneal macrophages with ISD and egg DNA for the indicated times and the cells were harvested for further immunoblotting of the activation status of TBK1 and IRF3. Consistent with previous reports, ISD induced the phosphorylation of TBK1 and IRF3, whereas phosphorylation of TBK1 and IRF3 was completely inhibited after *Cgas* KO (Fig 6H–6J). Moreover, deficiency of *Cgas* did not significantly affect LPS-induced phosphorylation of TBK1 and IRF3 (Fig 6K–6M). Notably, transfection of cells with either egg DNA or adult worm-derived DNA induced the phosphorylation of TBK1 and IRF3 (Fig 6H and 6K). In particular, phosphorylation of TBK1 and IRF3 induced by egg DNA was largely impaired when *Cgas* was knocked out (Fig 6H–6J). By contrast, the deletion of *Cgas* only partly reduced the phosphorylation of TBK1 and IRF3 in response to adult worm-derived DNA stimulation (Fig 6K–6M). These results suggested that cGAS might be an important recognition molecule for schistosome-derived DNA and is critical for the activation of downstream signaling pathways.

## Discussion

Multiple PRRs have been demonstrated to be involved in the regulation of the pathogenesis of schistosomiasis [6–12]. However, little is known about the role of DNA sensors in the regulation of *S*. *japonicum* infection. In this study, we revealed a detrimental role of the cGAS-STING axis in regulating *S*. *japonicum* infection, involving sensing of egg-derived DNA. Importantly, cGAS exacerbates *Schistosoma* infection by promoting the formation of granulomas and boosting liver fibrosis in both STING-dependent and -independent manners (S2 Fig).

A recent work demonstrated that STING deficiency leads to enhanced resistance to *S*. *mansoni* infection [28], which supports our findings. Mechanistically, in the context of *S*. *mansoni* infection, the deficiency of *Sting* resulted in a significant increase in IFN-γ production by spleen cells and an elevated percentage of neutrophils in the lungs, bronchoalveolar lavage, and spleen. Meanwhile, a microbiota composition with a more inflammatory profile was observed in *Sting*^-/-^ mice when compared with their wild-type (WT) counterparts [28]. Our results demonstrated that the STING-mediated type I IFN response might be important for the regulation of *S*. *japonicum* infection, providing an alternative and additional mechanism underlying the exacerbation role of STING in the pathogenesis of schistosomiasis. In contrast to the requirement of STING for the formation of egg granulomas in liver of mice infected with *S*. *japonicum*, *Sting*^-/-^ mice showed no change in granuloma numbers and area when compared to WT animals in response to *S*. *mansoni* infection, which might reflect the difference in the pathogenesis of schistosomiasis caused by *S*. *japonicum* and *S*. *mansoni* [3,39].

*S*. *japonicum* egg granuloma lesions are caused by soluble egg antigens (SEA) secreted by mature eggs in the host tissue [39]. The secreted SEA from trichomonads deposited in mature eggs in the host tissue is released through the microscopic pores of the eggshell and influence T-cell differentiation, which regulates the formation of egg granulomas [31,40]. Our work demonstrated that the cGAS-STING-type I IFN signaling axis is critical for the regulation of egg granuloma formation in the liver of schistosome-infected mice. Notably, the cGAS-STING pathway has been implicated as being critical for the regulation of the homeostasis of intestinal immunity [41–44]. Moreover, the intestine is an important organ for the deposition of eggs [45,46]. However, the exact role of cGAS-STING in the regulation of granuloma in the intestine of schistosome-infected mice requires investigation.

Cytokines are critical components involved in the pathogenesis of schistosomiasis [47]. To date, CD4^+^ T cell subsets have been classified into several distinct T helper (Th) phenotypes including Th1, Th2, Th17, T follicular helper cells (Tfh), Th9, and regulatory T cells (Tregs). In the case of schistosomiasis, the granulomatous inflammation and chronic liver pathology are critically regulated by Th1/Th2 responses. Animal studies suggest that there is a moderate Th1 response to parasite antigens during the acute stage, but then egg-derived antigens induce a sustained and dominant Th2 response that mediates granuloma formation and liver fibrosis [31]. Further studies have shown that Th2, Th9, and Th17 CD4^+^ T cells promote granuloma formation by secreting cytokines such as interleukin (IL)-4, IL-9 and IL-17A, respectively. Macrophages have also been demonstrated to contribute to granuloma formation by recruiting Tfh cells to the vicinity of the granuloma through cell-cell contact. In contrast, a variety of immune cells have been reported to inhibit granuloma formation. Th1 cells inhibit the granulomatous response by secreting IFN-γ. Treg cells can secrete IL-10 to suppress the granulomatous response. Thus, T lymphocytes and their associated cytokines play an important role in shaping the local microenvironment to regulate the pathological progression of schistosomiasis [40]. However, the function of IFNβ in regulating *Schistosoma* infection remains elusive. In the present study, we found that *S*. *japonicum* infection caused increased production of IFNβ in mice, with a peak at 4 weeks post infection. Importantly, the administration of IFNβ by tail vein injection markedly increased the pathological damage to the liver and facilitated the formation of egg granulomas. In the context of *S*. *mansoni* infection, type I IFN signaling has been demonstrated to be required for optimal dendritic cell (DC) activation initiating Th2 responses *in vivo* [48]. Therefore, whether IFNβ might promote egg granuloma formation by modulating DC activation and Th2 responses in the process of *S*. *japonicum* infection warrants further investigation.

Using an acute mouse infection model, we observed that deficiency of *Cgas* or *Sting* drastically reduced the production of IFNβ in both the liver and peripheral blood, indicating that the cGAS-STING axis is essential for the induction of the type I IFN response. Macrophages are sources of cytokines and have been demonstrated to be involved in the pathogenesis of schistosomiasis [35–37]. In this study, we demonstrated that depletion of macrophage by clodronate liposomes markedly reduced the level of *Ifnb1* transcripts in the liver and the abundance of serum IFNβ in mice infected with *S*. *japonicum*, indicating that macrophages might serve as an important cellular source for the production of IFNβ during schistosome infection. Consistently, DNA isolated from both egg and adult worms triggered a type I IFN response in macrophages. Importantly, deletion of *Cgas* markedly reduced the type I IFN responses, including the production of Ifnb1 and Cxcl10, as well the phosphorylation of TBK1 and IRF3, indicating that cGAS is required for the induction of type I IFN responses in response to schistosome-derived DNA in macrophages. A recent work revealed that *S*. *mansoni* DNA is sensed by cGAS leading to STING activation in murine embryonic fibroblasts (MEFs) [28], indicating that the sensing of parasite-derived DNA by cGAS is common to different species of *Schistosoma*. cGAS is a cytosolic DNA sensor, while helminths are different from intracellular parasites. On the one hand, during the process of *S*. *japonicum* infection, the deposited eggs might be phagocytosed by macrophages, thereby delivering DNA into the cytosol to activate cGAS in phagocytic cells. On the other hand, the stresses caused by infection might cause damage to cells, including macrophages, which might result in DNA damage or mitochondrial dysfunction. Such processes will lead to the accumulation of DNA, either from the nucleus or mitochondria, thereby triggering the cGAS-STING pathway. Therefore, during the natural infection of *Schistosoma*, cGAS-mediated sensing of parasite-derived DNA might be critical for the induction of the type I IFN response, at least in macrophages, which might ultimately contribute to the pathogenesis of schistosomiasis. Notably, bacteria-derived cyclic dinucleotides (CDNs), including c-di-AMP and c-di-GMP, have been reported to be critical for triggering STING-mediated immunity [49], thus it is tempting to speculate that schistosomes might produce CDNs to stimulate STING activation and thereby induce immunity against *Schistosoma* infection.

Hepatic fibrosis (HF) caused by granulomas of liver eggs is the main pathological change during schistosome infection [33]. The pathogenesis of liver fibrosis in schistosomiasis is the result of the combined participation of cellular and humoral immunity [47]. The egg antigens are eliminated slowly, and the persistent granulomatous response leads to prolonged matrix synthesis and HF, which is characterized by the proliferation of extracellular matrix (ECM) following liver injury. Essentially, the synthesis of ECM outweighs its degradation to the extent that excessive deposition of ECM leads to the replacement of liver parenchymal cells by scar tissue, eventually leading to cirrhosis. In recent years, a series of reports have shown that hepatic stellate cells (HSCs) are key effector cells in the formation of liver fibrosis and that upon activation, they can transform into myofibroblasts and secrete collagen to cause liver fibrosis. However, the exact mechanism underlying cGAS-mediated regulation of HF in the process of schistosome infection remains to be determined. Notably, the profibrotic effect of cGAS suggests caution regarding the potential harmful effects of targeting cGAS to prevent multiple type of diseases, including inflammatory diseases and cancer [20,50].

Early studies of cGAS focused on its classical function in regulating innate immunity. Activation of this signaling pathway plays an important role in the host response to infection by multiple types of pathogens, including viruses [51,52], bacteria including *Mycobacterium tuberculosis* [53], *Listeria monocytogenes* [54], and *Neisseria gonorrhoeae* [55], as well as parasites [22–27], which is largely dependent on STING. However, the novel functions of cGAS beyond sensing DNA and innate immunity have been extensively revealed in last decade. cGAS has been reported to be important for the regulation of cell senescence [56], cell apoptosis [57–59], autophagy [60], DNA repair [61,62], DNA replication [63], the stemness of T cells [64], and vascular neogenesis [65]. Currently, it is generally accepted that in addition to STING-dependent functions, cGAS also harbors STING-independent functions, such as inhibition of homologous recombination-mediated DNA repair [62], micronucleophagy [66], acting as a decelerator of replication forks [67], and promotion of replicative senescence [68]. Here, we demonstrated a novel function of cGAS in promoting liver fibrosis during schistosome infection, independent of STING, further proving the uncoupled functions of cGAS and STING in multiple biological processes.

In this study, we established a critical role of the cGAS-STING-type I IFN signaling axis in the regulation of egg granuloma formation and revealed an unexpected role of cGAS in regulating HF in a STING-independent manner. The findings further increase our understanding of the pathogenesis of schistosomiasis and provide a basis for the design of novel strategies to treat this disease.

## Material and methods

### Ethics statement

All animal experiments were performed in strict accordance with the Regulations for the Administration of Affairs Concerning Experimental Animals (approved by the State Council of People’s Republic of China), and efforts were made to minimize suffering. All procedures performed on animals in this study were approved by the Laboratory Animal Welfare & Ethics Committee (LAWEC) of the National Institute of Parasitic Diseases, Chinese Centre for Disease Control and Prevention (Chinese Center for Tropical Diseases Research) (approval ID: IPD 2019–12).

### Reagents

The phosphatase inhibitor cocktail and protease inhibitor cocktail were purchased from Sigma-Aldrich (St. Louis, MO, USA). The following antibodies were used: Anti-cGAS (#15102), anti-TBK1 (#3504S), anti-phospho-TBK1 (#5483), anti-IRF3 (#4302), anti-phospho-IRF3 (#4947), anti-α-Smooth Muscle Actin (D4K9N) (#19245), anti-GAPDH (D16H11) (#5174), Horseradish peroxidase (HRP)-conjugated goat anti-rabbit or anti-mouse IgG (all from Cell Signaling Technology, Danvers, MA, USA). Anti-Collagen I (ab6308)) and anti-Collagen III (ab184993) were both purchased from Abcam (Cambridge, MA, USA). The ReverTra Ace qPCR RT Kit (FSQ-101) and SYBR RT-PCR kit (QPK-212) were purchased from Toyobo (Osaka, Japan). LPS was purchased from Sigma-Aldrich. Immunostimulatory DNA (ISD, TACAGATCTACTAGTGATCTATGACTGATCTGTACATGATCTACA) was purchased from Invivogen (San Diego, CA, USA).

### Mice, parasites, and infection

Six to eight-week-old, female wild-type C57BL/6 mice were purchased from the Shanghai SLAC Laboratory Animal Co., Ltd (Shanghai, China). *Cgas* and *Sting* knockout mice with a C57BL/6 background were purchased from the Jackson Laboratories (Bar Harbor, ME, USA) and were housed and bred in specific pathogen free class animal houses. Female New Zealand White rabbits (6–8 weeks old), were purchased from Shanghai Songlian Experimental Animal Farm (Shanghai, China) and were housed in the rabbit house of the Institute Experimental Animal Centre.

WT mice, *Cgas* knockout mice, and *Sting* knockout mice were randomly selected for infection with *S*. *japonicum*. Mice were each infected percutaneously via the shaved skin of the abdomen, with 20 ± 2 *S*. *japonicum* cercariae obtained from the National Institute of Parasitic Diseases, Chinese Center for Disease Control and Prevention (Shanghai, China).

### Depletion of macrophages in mice using clodronate liposomes

The depletion of macrophages in mice using clodronate liposomes was performed as described previously [38]. Mice were intraperitoneally injected with 200 μL of control liposomes or clodronate liposomes twice a week at 1 week before infection, and administered continuously at an interval of twice a week during the infection process.

### Isolation of mouse peritoneal macrophages

Six to eight-week-old wild-type and *Cgas* knockout C57BL6 mice were injected intraperitoneally with 2 ml of 4% thioglycollate (Becton Dickinson Company, Franklin Lakes, NJ, USA). After three days, the mice were sacrificed the peritoneal cavity was cut open with scissors, and 10 ml of Roswell Park Memorial Institute (RPMI)-1640 medium was injected into the peritoneal cavity using a 10 ml syringe (a 1 ml syringe needle was used) and the mice were lightly shaken. The contents of the mouse peritoneal cavity were then recovered from the peritoneum using a 10 ml syringe (with a 10 ml syringe needle) and collected in a 50 ml sterile centrifuge tube. The cells were centrifuged at room temperature (1200 × *g* for 3–5 minutes), resuspended in complete RPMI-1640 medium, and seeded in plates. After 2–4 hours, at which point the adherent cells are mainly mouse peritoneal primary macrophages, the medium was replaced with fresh complete medium. The cells were maintained at 37°C in 5% CO_2_ and applied for further experiments.

### Purification of *S*. *japonicum* egg and adult worm DNA

The livers of New Zealand White rabbits infected with *S*. *japonicum* for 7 to 8 weeks were harvested for the extraction of eggs using a method modified from a previous report [69]. The adult worms were also collected. The DNA of eggs and adult worms was extracted according to the manual of the genomic DNA extraction kit (D0065S, Beyotime Technology, Shanghai, China).

### Stimulation of mouse peritoneal macrophages

1.5× 10^6^ wild-type and *Cgas* knockout mouse peritoneal macrophages were seeded in a 6-well-plate followed by the indicated stimulations. 2 μg ISD or DNA purified from *S*. *japonicum* eggs and adult worms was applied for the transfection of macrophages using Lipofectamine 2000 reagent (ThermoFisher Scientific). Briefly, 2 μg DNA and 4 μL Lipofectamine 2000 were diluted in 100 μL Opti-MEM medium, separately. Then the DNA and Lipofectamine 2000 dilutions were mixed and incubated at room temperature for 5 min. The DNA-lipid complex was then added to the cell culture dropwise for stimulation. LPS (1 μg/mL) was directly added into the cell culture (1.5×10^6^) and kept for indicated times for the stimulation. The cells were then harvested for further analysis.

### Western blotting

Cells or tissues were lysed using Radioimmunoprecipitation assay (RIPA) Lysis Buffer (Beyotime Biotechnology, China) supplemented with protease inhibitor cocktail (P8340, Sigma-Aldrich), 1 mM of phenylmethylsulfonyl fluoride (PMSF), and a phosphatase inhibitor cocktail (P5726, Sigma-Aldrich). The lysates were centrifuged at 10,000 × *g* for 10 min and the cellular debris was discarded. Proteins were loaded on 4–20% Mini-PROTEAN TGX Precast Gels (Bio-Rad, Hercules, CA, USA). The separated proteins were then transferred onto nitrocellulose membranes. The membranes were blocked in 5% skim milk and probed with the indicated antibodies at a dilution of 1:1000. Detection was performed using a Chemiluminescent Substrate (Pierce, Thermo Fisher Scientific, Waltham, MA, USA) and the immunoreactive protein bands visualized on a Fusion FX imaging system (Vilber, Collégien, France).

### Quantitative real-time reverse transcription PCR (qRT-PCR)

Total RNA was extracted from liver tissue using TRIzol (Invitrogen, Carlsbad, CA, USA). For each sample, 2 μg of total RNA was reverse transcribed using a complementary DNA (cDNA) reverse transcription kit (Takara, Dalian, China). The cDNA was then subjected to qPCR. The comparative threshold cycle (2^−ΔΔCt^) method was used to evaluate the relative mRNA expression, and *Gapdh* (glyceraldehyde-3-phosphate dehydrogenase) levels were used as a normalization control. The primers used in this study are listed in **Table 1**.

**Table 1:** Primer sequences used in qRT-PCR analysis.

Gene	Forward (5′–3′)	Reverse (5′–3′)
*Gapdh*	GAGCCAAACGGGTCATCATCT	GAGGGGCCATCCACAGTCTT
*Ifnb1*	AGCTCCAAGAAAGGACGAACA	GCCCTGTAGGTGAGGTTGAT
*Cxcl10*	CCAAGTGCTGCCGTCATTTTC	GGCTCGCAGGGATGATTTCAA
*Cgas*	CCAAGCTGGTCTACCACCTG	GCGGTTCCTGCACTTCAAC
*Sting*	GGTCACCGCTCCAAATATGTAG	CAGTAGTCCAAGTTCGTGCGA

### cGAMP quantitation assay

The liver tissues were collected from mice left uninfected or infected with *S*. *japonicum* for indicated times, and the homogenates were centrifuged at 12 000 × g for 5 min and the supernatants were applied for the cGAMP detection using a cGAMP Enzyme Immunoassay Kit (K067-H1, Arbor Assays, Ann Arbor, MI, USA).

### ELISA

Peripheral blood was collected from WT and *Cgas* KO or *Sting* KO mice post infection via the orbital plexus. The collected blood was left to stand at room temperature for 2 h and was then centrifuged at 2 000 × *g* for 15 min. The supernatants were harvested as serum and stored at **-**80°C. The levels of IFNβ in the serum were measured using a Mouse IFNβ Quantikine ELISA Kit, according to the manufacturer’s instructions (#MIFNB0, R&D Systems Inc., Minneapolis, MN, USA).

### Histological analysis

Liver lobes of mice infected with *S*. *japonicum* were fixed in 4% paraformaldehyde for subsequent experiments. The fixed tissues were then embedded in paraffin and cut into serial sections. H&E staining was used to detect pathological damage and oval granulomas. Masson staining was performed for the analysis of liver fibrosis. The histological analysis was done by Wuhan Saville Biologicals (Wuhan, China) and five noncontinuous sections of each liver sample were tested. The granuloma size was quantified using CaseViewer 2 software (3DHISTECH, Budapest, Hungary).

### Statistical analysis

All quantitative data were reported as the mean and standard error of the mean (SE). All samples were compared using an unpaired Student’s *t*-test, one-way analysis of variance (ANOVA), or two-way ANOVA. The survival rate was analyzed using the Kaplan–Meier method, and the difference between survival curves was tested for statistical significance using the log-rank test. *p* < 0.05 was considered to indicate statistical significance. GraphPad Prism 6 (GraphPad Software Inc., San Diego, CA, USA) was used for all statistical analyses and graph preparation.

All numerical values that were used to generate graphs and histograms are included in S1 Data. The source data for the immunoblotting in the figures are included in S2 Data.

## Supporting information

S1 Fig cGAS is required for an ISD, but not an LPS, -induced type I IFN response. (A-B) qRT-PCR measurement of transcripts of *Ifnb1* and *Cxcl10* in wild-type and *Cgas* knockout peritoneal macrophages transfected with ISD for the indicated times. (C-D) qRT-PCR measurement of transcripts of *Ifnb1* and *Cxcl10* in wild-type and *Cgas* knockout peritoneal macrophages stimulated with LPS for the indicated times. Two-way ANOVA with a Bonferroni’s post hoc test were used for the statistical analysis. ns, not significant; *, *p* < 0.05, **, *p <* 0.01, ***, *p <* 0.001.
(TIFF)

S2 Fig Diagram showing the functional role of cGAS-STING in the process of *Schistosoma japonicum* infection. During natural infection of *S*. *japonicum*, the parasite-derived DNA, such as egg DNA, might be sensed by cGAS, which then catalyzes the formation of cGAMP. The generated cGAMP or parasite-derived CDNs might be sensed by STING, which then activates TBK1-IRF3 and subsequently induces the type I IFN response, which promotes granuloma formation. Intriguingly, cGAS also exacerbates liver fibrosis in response to *S*. *japonicum* infection in a STING-independent manner.
(TIFF)

S1 Data Excel spreadsheet containing, in separate sheets, the numerical data for figure panels Figs 1B, 1C, 1E, 1F, 1G, 1I, 1K, 1M, 1N, 1O, 2B, 2C, 2E, 2F, 2G, 2I, 2K, 2M, 2N, 3A, 3B, 3C, 3D, 3E, 3F, 4B, 4D, 4E, 4F, 4G, 4H, 4J, 5A, 5B, 5D, 5E, 5F, 5H, 6B, 6C, 6D, 6E, 6F, 6G, 6I, 6J, 6L, 6M, and S1A, S1B, S1C, S1D. (XLSX)

S2 Data The source data for the immunoblotting shown in figure panels Figs 1L, 2L, 5C, 5G, 6H and 6K. (PDF)
