# Supplementary figures and images for: cGAS exacerbates Schistosoma japonicum infection in a STING-type I IFN-dependent and independent manner
Source: PLoS Pathog. 2022 Feb 2;18(2):e1010233. doi: 10.1371/journal.ppat.1010233 (PMC8809611; doi:10.1371/journal.ppat.1010233)

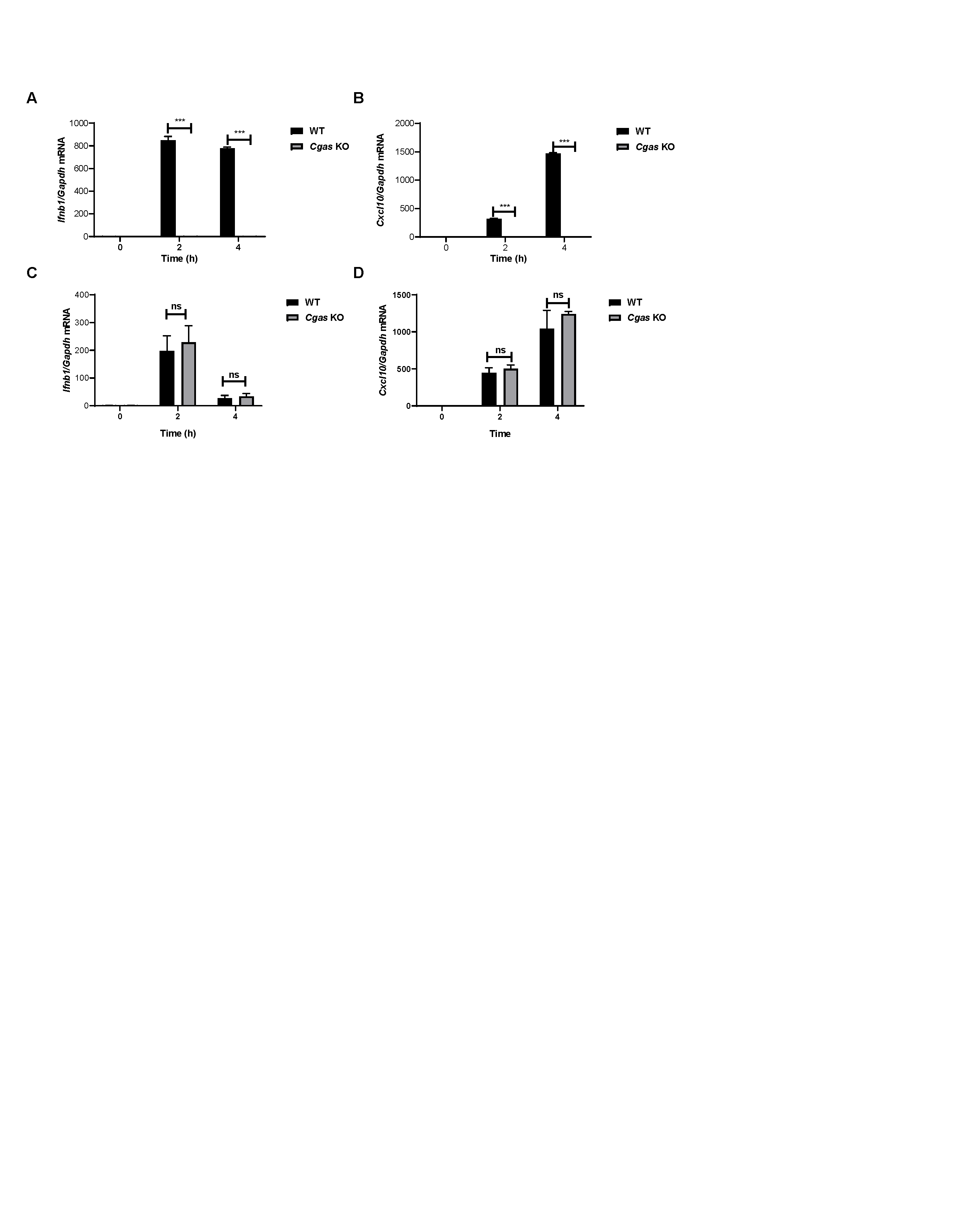

Supplement: S1 Fig — (A-B) qRT-PCR measurement of transcripts of Ifnb1 and Cxcl10 in wild-type and Cgas knockout peritoneal macrophages transfected with ISD for the indicated times. (C-D) qRT-PCR measurement of transcripts of Ifnb1 and Cxcl10 in wild-type and Cgas knockout peritoneal macrophages stimulated with LPS for the indicated times. Two-way ANOVA with a Bonferroni’s post hoc test were used for the statistical analysis. ns, not significant; *, p < 0.05, **, p < 0.01, ***, p < 0.001. (TIFF) [file ppat.1010233.s001.tiff]

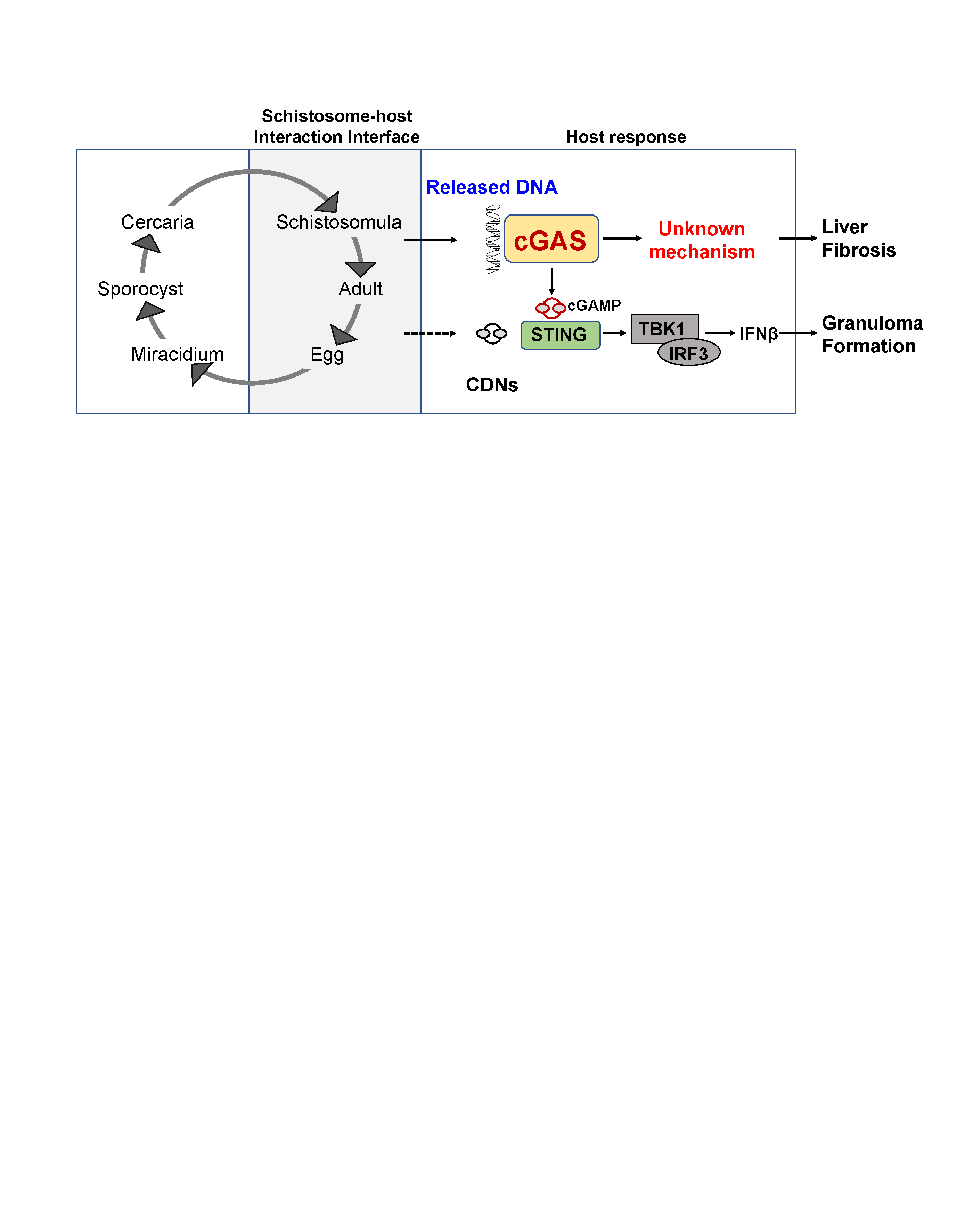

Supplement: S2 Fig — During natural infection of S. japonicum, the parasite-derived DNA, such as egg DNA, might be sensed by cGAS, which then catalyzes the formation of cGAMP. The generated cGAMP or parasite-derived CDNs might be sensed by STING, which then activates TBK1-IRF3 and subsequently induces the type I IFN response, which promotes granuloma formation. Intriguingly, cGAS also exacerbates liver fibrosis in response to S. japonicum infection in a STING-independent manner. (TIFF) [file ppat.1010233.s002.tiff]

Figure 1L

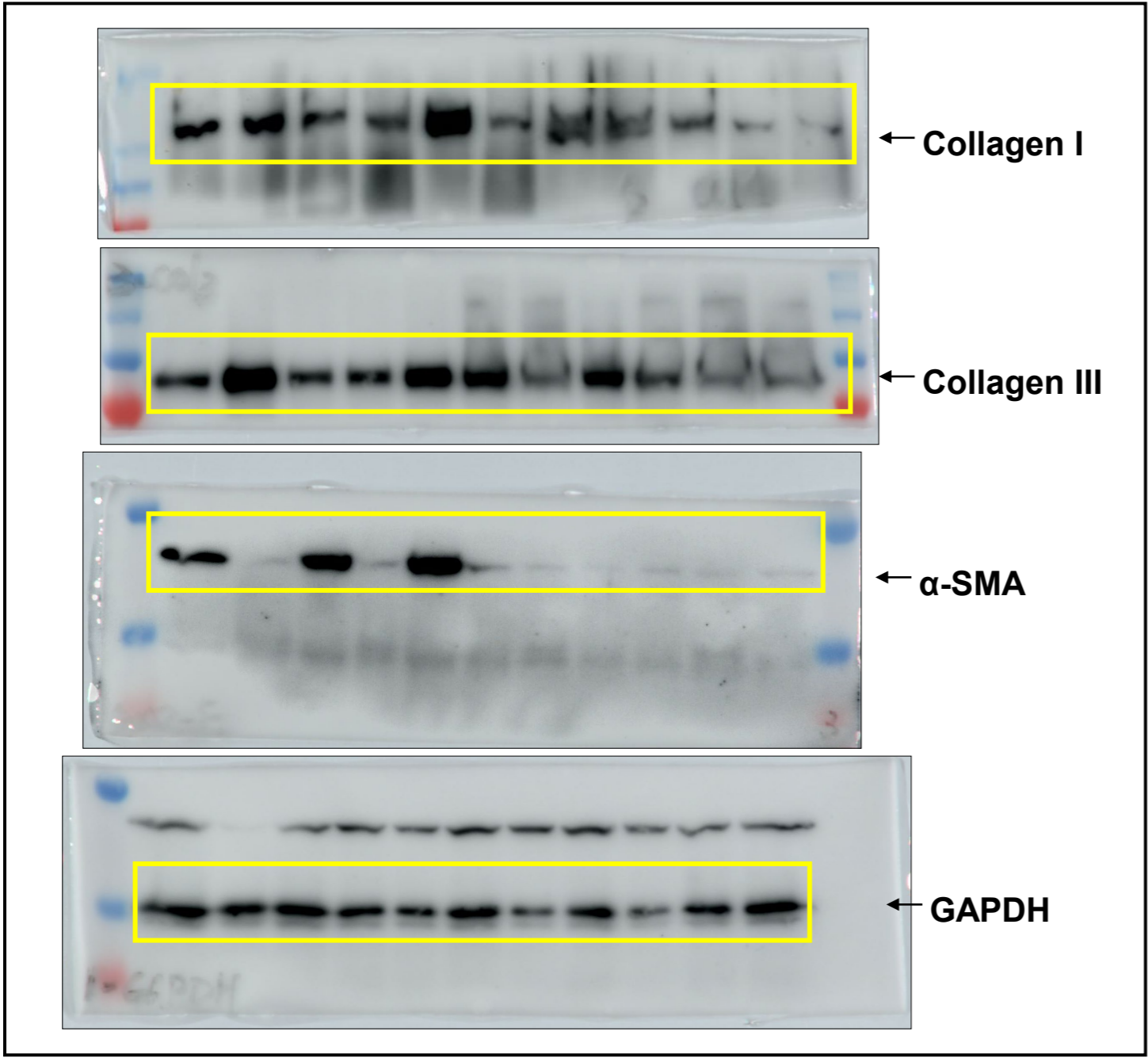

Figure 2L

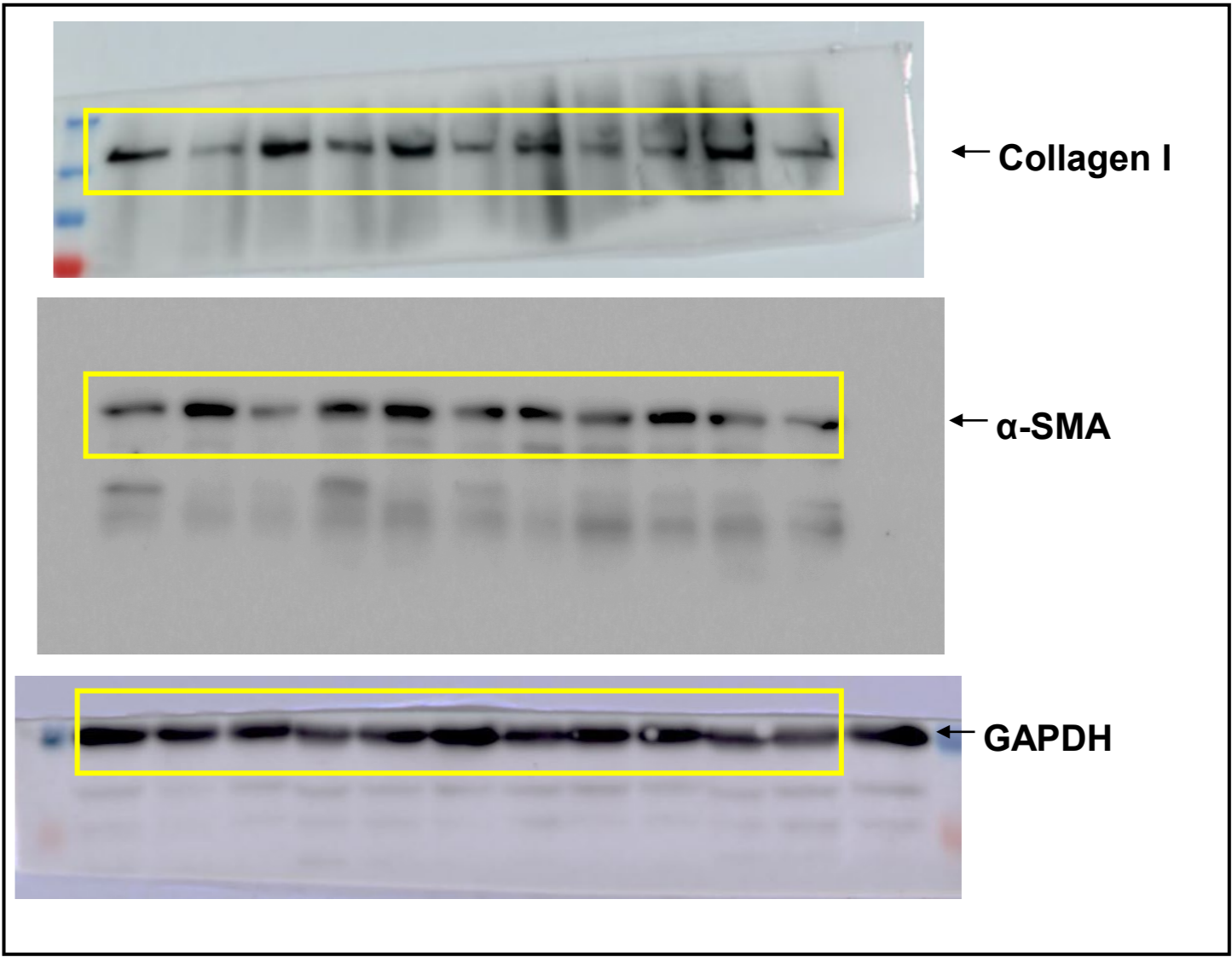

Figure 5C

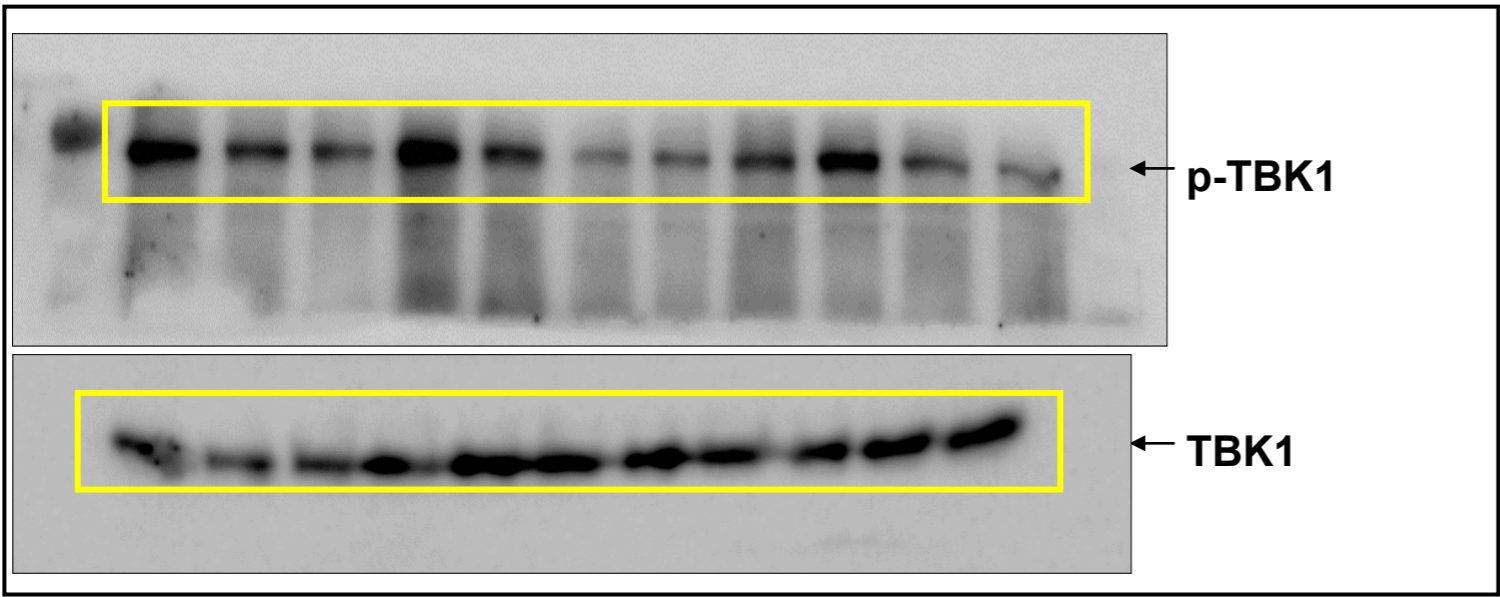

Figure 5G

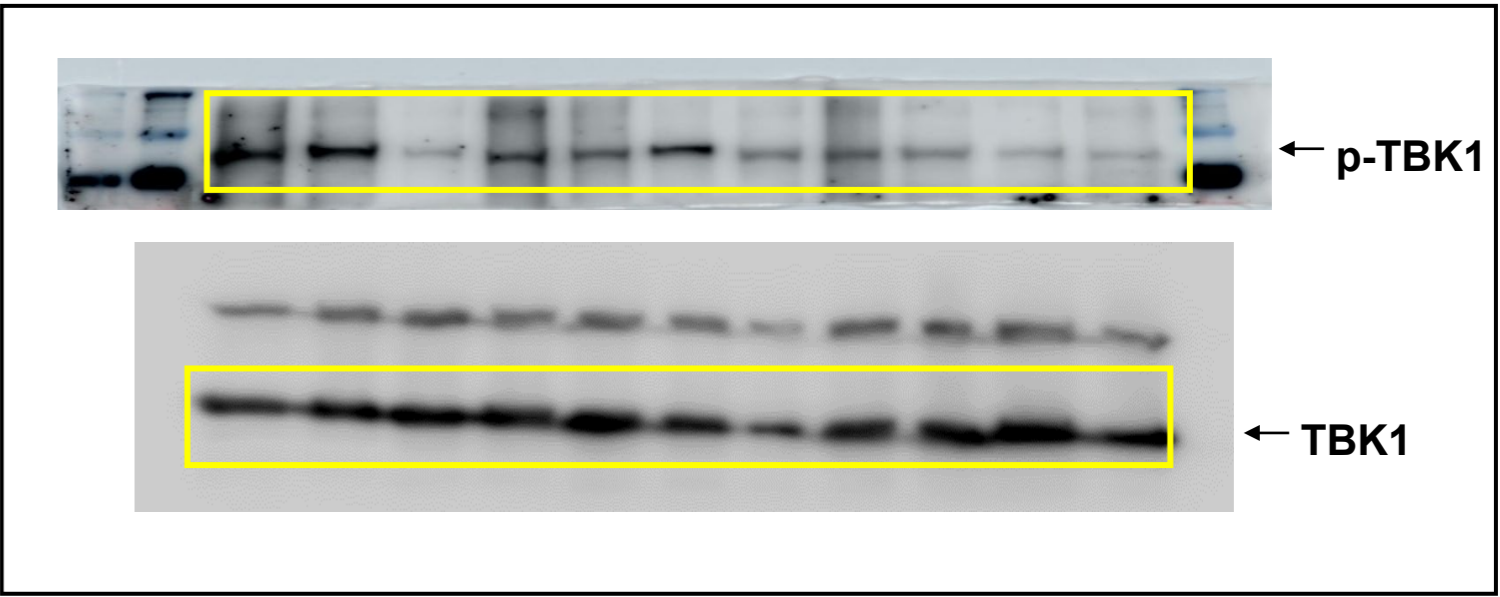

Figure 6H

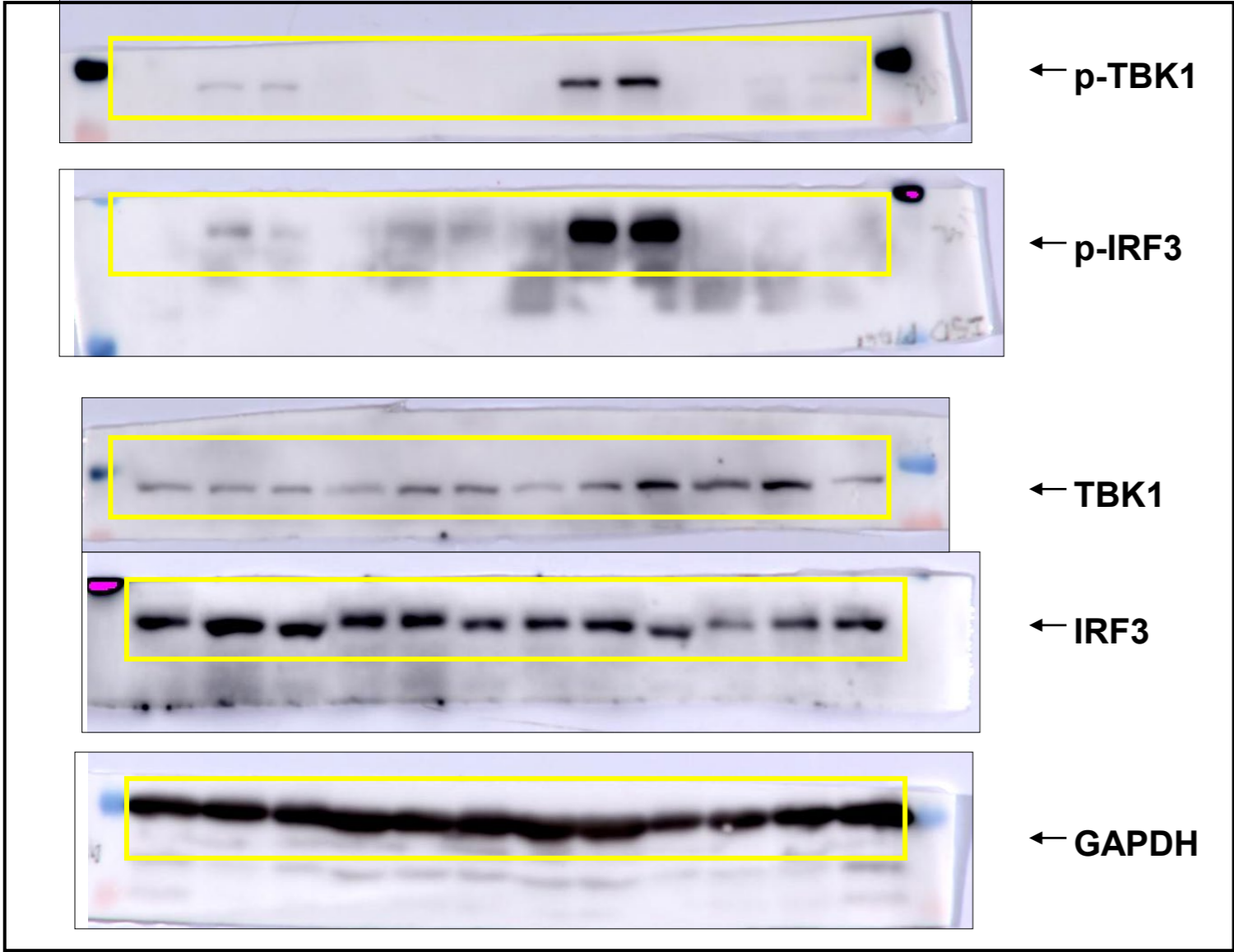

Figure 6K

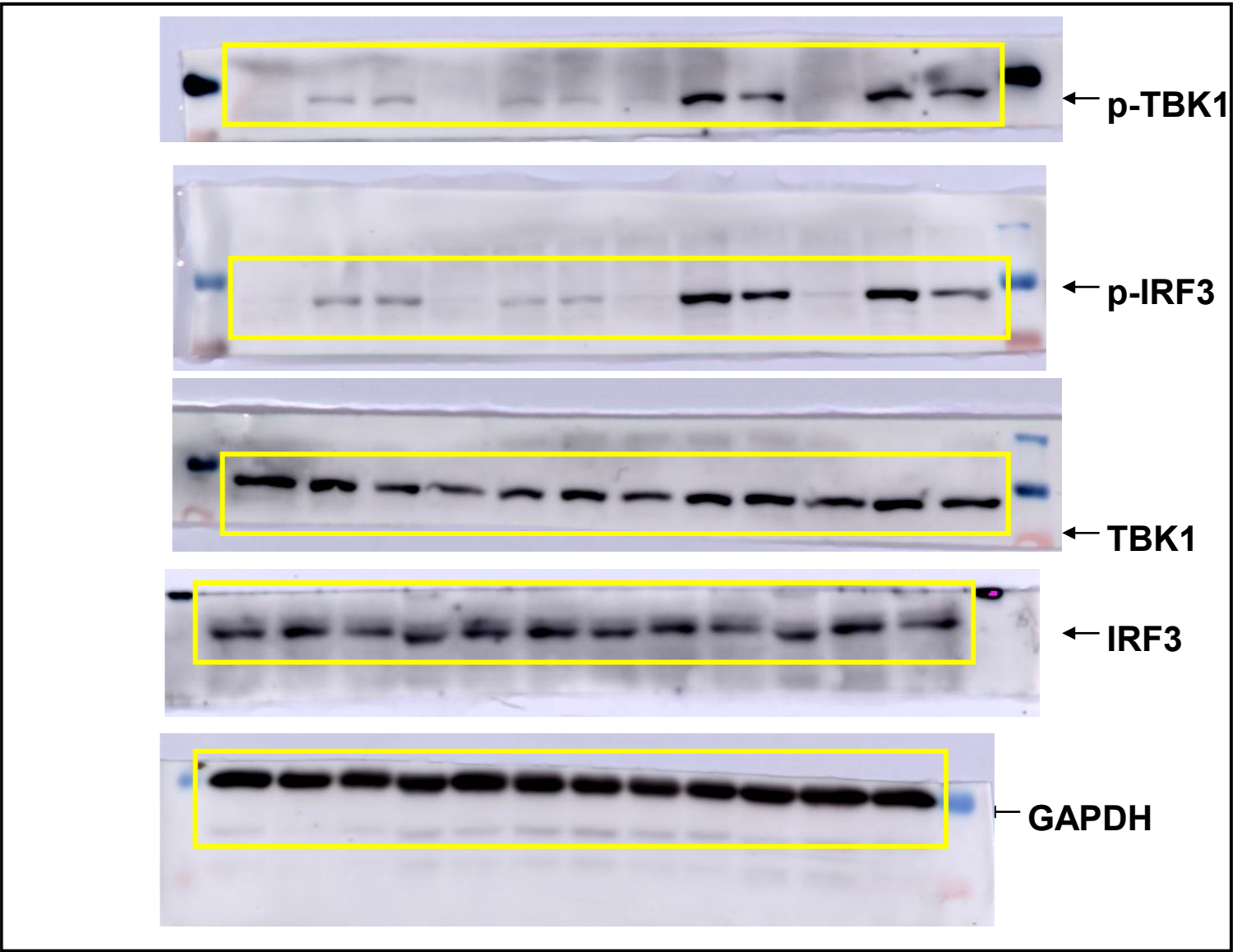

Supplement: S2 Data — (PDF) [file ppat.1010233.s004.pdf]
